# Diagnosis and management of X-linked hypophosphatemia in children and adolescent in the Gulf Cooperation Council countries

**DOI:** 10.1007/s11657-021-00879-9

**Published:** 2021-03-04

**Authors:** Fahad Al Juraibah, Elham Al Amiri, Mohammed Al Dubayee, Jamal Al Jubeh, Hessa Al Kandari, Afaf Al Sagheir, Adnan Al Shaikh, Salem A. Beshyah, Asma Deeb, Abdelhadi Habeb, Manal Mustafa, Hanaa Zidan, M. Zulf Mughal

**Affiliations:** 1 Department of Paediatrics, Ministry of the National Guard – Health Affairs, Riyadh, Saudi Arabia; 2 College of Medicine, King Saud bin Abdulaziz University for Health Science, Riyadh, Saudi Arabia; 3 Al Qassimi Women & Children Hospital, Sharjah, United Arab Emirates; 4 Division of Paediatric Endocrinology, Institute of Paediatrics, Sheikh Khalifa Medical City, Abu Dhabi, United Arab Emirates; 5 Farwaniya Hospital, Kuwait City, Kuwait; 6 Department of Population Health, Dasman Diabetes Institute, Kuwait City, Kuwait; 7 King Faisal Specialist Hospital & Research Centre, Riyadh, Kingdom of Saudi Arabia; 8 King Abdulaziz Medical City, Ministry of the National Guard, Jeddah, Saudi Arabia; 9 Diabetes and Endocrine Clinic, Mediclinic Airport Road, Abu Dhabi, United Arab Emirates; 10 Department of Medicine, Dubai Medical College for Girls, Dubai, United Arab Emirates; 11 Division of Paediatric Endocrinology, Sheikh Shakhbout Medical City, Abu Dhabi, United Arab Emirates; 12 Department of Paediatrics, Prince Mohammed bin Abdulaziz Hospital for National Guard, Al-Madinah, Saudi Arabia; 13 Latifa Women & Children Hospital, Dubai Health Authority, Dubai, United Arab Emirates; 14 American Hospital, Dubai, United Arab Emirates; 15 Royal Manchester Children’s Hospital, Manchester University Hospitals NHS Foundation Trust, Oxford Road, Manchester, M13 9WL UK; 16 Faculty of Biology, Medicine & Health, University of Manchester, Manchester, UK

**Keywords:** Rickets, Osteomalacia, Hypophosphatemia, X-linked hypophosphatemia (XLH), Fibroblast growth factor-23 (FGF23), Burosumab, Gulf Cooperation Council (GCC) countries

## Abstract

**Introduction:**

X-linked hypophosphatemia (XLH) is a rare inherited cause of hypophosphatemic rickets and osteomalacia. It is caused by mutations in the phosphate-regulating endopeptidase homolog, X-linked (PHEX). This results in increased plasma fibroblast growth factor-23 (FGF23), which leads to loss of renal sodium-phosphate co-transporter expression leading to chronic renal phosphate excretion. It also leads to low serum 1,25-dihydroxyvitamin D (1,25(OH)_2_D), resulting in impaired intestinal phosphate absorption. Chronic hypophosphatemia in XLH leads to impaired endochondral mineralization of the growth plates of long bones with bony deformities. XLH in children and adolescents also causes impaired growth, myopathy, bone pain, and dental abscesses. XLH is the most frequent inherited cause of phosphopenic rickets/osteomalacia. Hypophosphatemia is also found in calcipenic rickets/osteomalacia as a result of secondary hyperparathyroidism. Thus, chronic hypophosphatemia is a common etiologic factor in all types of rickets.

**Results:**

There is considerable overlap between symptoms and signs of phosphopenic and calcipenic rickets/osteomalacia. Wrong diagnosis leads to inappropriate treatment of rickets/osteomalacia. Nutritional rickets and osteomalacia are common in the Gulf Cooperation Council countries which include Saudi Arabia, United Arab Emirates, Kuwait, Qatar, Bahrain, and Oman. Due to high levels of consanguinity in the region, genetic causes of phosphopenic and calcipenic rickets/osteomalacia are also common.

**Conclusion:**

This guideline was developed to provide an approach to the diagnosis of XLH, especially where there is no family history of the disease, and that other related conditions are not mistaken for XLH. We also guide the medical management of XLH with conventional treatment and with burosumab, a recombinant human IgG1 monoclonal antibody to FGF23.

## Pathophysiology of rickets and osteomalacia

Rickets is a bone disease of a growing child in which there is a failure of maturation and mineralization of the growth plate and osteoid matrix, resulting from conditions that cause chronically low concentrations of calcium and phosphate in the extracellular fluid. The orderly differentiation of the growth plate is regulated by several growth and transcription factors. Chondrocytes in the “resting zone” of the growth plate, adjacent to the epiphysis, undergo a sequence of maturation processes; apoptosis of terminally differentiated hypertrophic chondrocytes, which is induced by phosphate ions in extracellular fluid, is a crucially important step for endochondral mineralization [1, 2].

Traditionally, rickets has been classified into “calcipenic rickets” and “phosphopenic rickets.” Calcipenic rickets is caused mainly by inadequate dietary calcium and/or vitamin D deficiencies, resulting in insufficient intestinal absorption of calcium. It also arises from defects in the metabolism of vitamin D which are due to either failure to synthesize 25-hydroxyvitamin D (25OHD) in vitamin D–dependent rickets type IB (VDDRIB) or failure to synthesize 1,25-dihydroxyvitamin D (1,25(OH)_2_D) in VDDRIA and end-organ resistance to its active metabolite 1,25(OH)_2_D as in VDDR2A and 2B (Table 1). In calcipenic rickets, a fall in serum calcium level leads to elevated plasma parathyroid hormone (PTH) level. Resulting secondary hyperparathyroidism causes rapid internalization and degradation of sodium-dependent phosphate co-transporter proteins (NaPi-2a and NaPi-2c) in the proximal renal tubules leading to renal phosphate excretion and hypophosphatemia. Phosphopenic rickets result from impaired dietary phosphate absorption or from causes that result in chronic renal phosphate wastage. Conditions that result in increased plasma levels or expression of the fibroblast growth factor-23 (FGF23) or inactivating mutations in genes encoding for sodium-dependent phosphate transporter in the proximal renal tubule result in increased urinary phosphate wastage. Chronic hypophosphatemia in calcipenic and phosphopenic rickets results in impaired apoptosis of terminally differentiated chondrocytes and failure of the growth plate’s mineralization. Thus, chronic hypophosphatemia is considered to be the unifying pathophysiological pathway in all types of rickets [1, 2]. Chronic hypophosphatemia also results in impaired mineralization of osteoid in children with rickets and older adolescents and adults with osteomalacia [3, 4].

**Table 1:** Laboratory characteristics of inherited and acquired causes of calcipenic and phosphopenic rickets and osteomalacia

Disease	MIM no.	Genetic defect	Plasma FGF23	TmP/GFR	Serum Ca	Serum P	Serum ALP	Plasma PTH	Serum 25OHD	Serum 1,25(OH)_2_D	Urine Ca excretion, urine Ca/Cr
Nutritional rickets caused by vitamin D and/or dietary calcium deficiency	N/A	N/A	N or ↓	↓	↓ or N	↓	↑↑↑	↑↑↑	↓↓↓ or N	N or ↓	↓
Vitamin D–dependent rickets type 1A (VDDR1A)	264700	CYP27B1	N or ↓	↓	↓↓	↓	↑↑↑	↑↑↑	N	↓↓	↓
Vitamin D–dependent rickets type 1B (VDDR1B)	600081	CYP2R1	N or ↓	↓	↓↓	↓	↑↑↑	↑↑↑	↓↓	↓	↓
Vitamin D–dependent rickets type 2A (VDDR2A)	277440	VDR	N or↓	↓	↓↓	↓	↑↑↑	↑↑↑	N	↑↑↑	↓
Vitamin D–dependent rickets type 2B (VDDR2B)	164020	HNRNPC	N or ↓	↓	↓↓ or N	↓	↑↑↑	↑↑↑	N	↑↑↑	↓
Vitamin D–dependent rickets type 3 (VDDR3)	N/A	CYP3A4	N or ↓	↓	↓↓	↓	↑↑↑	↑↑↑	↓↓	↓	↓
X-linked hypophosphatemia (XLH)	307800	PHEX	↑or N	↓	N	↓	↑↑	N or ↑	N	N or ↓	N or ↓
Autosomal dominant hypophosphatemic rickets (ADHR)	193100	FGF23	↑or N	↓	N	↓	↑↑	N or ↑	N	N or ↓	N or ↓
Autosomal recessive hypophosphatemic rickets 1 (ARHR1)	241520	DMP1	↑or N	↓	N	↓	↑↑	N or ↑	N	N or ↓	N or ↓
Autosomal recessive hypophosphatemic rickets 2 (ARHR2)	613312	ENPP1	↑or N	↓	N	↓	↑↑	N or ↑	N	N or ↓	N or ↓
Autosomal recessive hypophosphatemic rickets 3 -Raine syndrome (ARHR3)	259775	FAM20C	↑or N	↓	N	↓	↑↑	N or ↑	N	N or ↓	N or ↓
Tumor-induced osteomalacia (TIO)	N/A	N/A	↑↑↑	↓	N	↓	↑↑	N or ↑	N	N or ↓	N or ↓
Cutaneous skeletal hypophosphatemia syndrome (SFM)	163200	RAS	↑ or N	↓	N	↓	↑↑	N or ↑	N	N or ↓	N or ↓
Osteoglophonic dysplasia (OGD)	66250	FGFR1	↑ or N	↓	N	↓	↑↑	N or ↑	N	N or ↓	N or ↓
Hypophosphatemic rickets with hypercalciuria (HHRH)	241530	SLC34A3	↓	↓	N	↓	↑↑	N	N	↑↑	↑
Hypophosphatemia and nephrocalcinosis (NPHLOP1)	612286	SLC34A1	↓	↓	N	↓	↑↑	N	N	↑	↑
Renal Fanconi syndrome, e.g., cystinosis	Various	Various mutations	↓ or N	↓	↓		↑↑	N or ↑	N	N or ↑	N or ↑
Phosphopenic rickets due to dietary phosphate deficiency or malabsorption	N/A	N/A	↓	↑	↓		↑↑	N	N	N	N

*N* normal, *↑* elevated, *↓ ↑* reduced, *N/A* information not available or not assessed, *MIM* Mendelian Inheritance in Man, *Ca* serum calcium corrected for albumin, *P* serum phosphate, *serum ALP* serum alkaline phosphatase activity, *TmP/GFR* renal tubular threshold maximum for phosphate, *plasma FGF23* fibroblast growth factor-23, *serum 25OHD* 25-hydroxyvitamin D, *1,25(OH)*_*2*_*D* 1,25-dihydroxyvitamin D (calcitriol), *urine Ca/Cr ratio* urine for calcium or creatinine ratio

## X-linked hypophosphatemia

X-linked hypophosphatemia (XLH) is a rare, multisystem genetic disorder characterized by hypophosphatemia secondary to chronic renal phosphate excretion. It is the most commonly inherited hypophosphatemic disorder with an estimated incidence of between 1:20,000 and 1:60,000 [5–7]. It is caused by loss of function mutations of the phosphate-regulating endopeptidase homolog, X-linked (PHEX), which results in an increased plasma concentration and expression of FGF23 [8, 9]. Raised plasma FGF23 reduces renal phosphate reabsorption by downregulating sodium/phosphate co-transporters NPT2a and NPT2c in the proximal renal tubules, resulting in increased urinary phosphate excretion and hypophosphatemia. Raised plasma FGF23 also suppresses the production of 1,25-dihydroxyvitamin D (1,25(OH)_2_D) in the proximal renal tubules. It does this by downregulating CYP27B1 (1α-hydroxylase; the enzyme that converts 25-hydroxyvitamin D (25OHD) to 1,25(OH)_2_D) and increasing its degradation by upregulating CYP24A1 (24-hydroxylase; enzyme responsible for the degradation of 25OHD and 1,25(OH)_2_D). Low or inappropriately normal serum 1,25(OH)_2_D for the prevailing serum phosphate level leads to impaired intestinal phosphate absorption [10]. Chronic hypophosphatemia resulting from increased renal phosphate wastage and reduced intestinal phosphate absorption results in failure of apoptosis of terminally differentiated hypertrophic chondrocytes and impaired mineralization of the growth plate.

Clinical manifestations of XLH vary in severity, even among affected individuals in the same kindred. Children with XLH develop genu varum or valgum upon weight-bearing, widening of ends of long bones, abnormal head shape due to craniosynostosis, progressive and disproportionate decline in linear growth, muscle weakness, bone pain, and dental abscesses due to impaired mineralization of enamel and dentine [3, 8, 11, 12]. Adolescents and adults with XLH develop pseudofractures and often complain of osteomalacia symptoms such as pain, fatigue, and impaired mobility. As the diseases progress, adults may experience debilitating sequelae of XLH, including deafness, osteoarthritis, enthesopathies, and spinal stenosis. Besides craniosynostosis, patients with XLH may develop motor and sensory and neurological symptoms secondary to Chiari type 1 malformation and associated syringomyelia.

## Why an XLH guideline for Gulf Cooperation Council (GCC) countries is needed?

XLH management in GCC countries urges for a consensus between the clinicians or guidelines based on multiple regional challenges. There are several clinical guidelines on the diagnosis and management of XLH provided by experts in the USA, France, and Japan [3, 6, 12]. More recently, Haffner and his European colleagues have produced an evidence-based consensus guideline based on systematic literature review and expert opinion [11]. Despite the availability of these experts and evidence-based guidelines, we are aware of cases in which the diagnosis of XLH in the GCC countries was delayed, particularly in patients with no family history of the disease. This delay may, in part, be due to the rarity of XLH, its variable severity, and multisystem involvement. In the absence of a multidisciplinary approach in dealing with complex cases, XLH among three generations in one family was missed and late-diagnosed despite serious skeletal malformations [13]. Since all cases of rickets have low serum phosphate levels, we are also aware that some XLH cases in the region have been misdiagnosed as nutritional rickets [14, 15]. This is not surprising as there is considerable clinical overlap between the various types of rickets and osteomalacia. Furthermore, cases of vitamin D deficiency as well as rickets and osteomalacia are common in the GCC countries, despite year-long abundance sunshine [16–18]. Due to the ongoing high levels of consanguinity in the region [19–22], recessively inherited conditions giving rise to calcipenic and phosphopenic rickets have also been misdiagnosed as XLH. Peter et al. reported a patient who as a child was diagnosed and managed as XLH and was correctly diagnosed in adulthood as VDDR types IA (biallelic mutation in the CYP27B1 gene), using the whole-exome sequencing [23]. Another report from Saudi Arabia highlights the difficulty in reaching the genetic diagnosis for VDDRIA and misdirection toward hypophosphatemic rickets in the genetic report [24]. Hypophosphatemic osteomalacia with raised plasma FGF23 level and no family history of rickets/osteomalacia was described in a female adult patient. Tumor-induced osteomalacia (TIO) was suspected; however, no tumor could be identified on extensive localization studies. Mutational analysis of the PHEX coding region and 3′UTR revealed the patient to be heterozygous for a novel germline PHEX mutation [25].

Wrong diagnosis leads to inappropriate treatment of rickets/osteomalacia. Misdiagnosis of other conditions that cause rickets/osteomalacia as XLH appears to be occurring despite educational activities aimed at increasing knowledge about XLH and its clinical manifestations, among health care providers in the GCC countries. Therefore, these guidelines were developed by 11 pediatric and an adult endocrinologist from the GCC countries, with expertise in managing bone disorders, who met on 21st June 2019. The writing group was convened under the auspices of the Arab Society of Paediatric Endocrinology and Diabetes (ASPED). The discussions were facilitated by an expert in pediatric bone diseases from the UK. The primary aim was to address challenges in the diagnosis of XLH in the region and to ensure that other rachitic conditions are not mistaken for XLH. The current guideline is to be used in connection with previously published expert and evidence-based XLH consensus guidelines [3, 6, 11, 12].

We also provide guidance on medical management of XLH in children and adolescents with oral phosphate and active vitamin D analogs, known as “conventional treatment,” and with burosumab, a recombinant human IgG1 monoclonal antibody to FGF23.

## An approach to the diagnosis of XLH

A detailed medical, including the family history, auxology, and musculoskeletal examination, and appropriate investigation (radiology, biochemistry, and genetic studies) helps to establish XLH diagnosis. More importantly, this approach can help to exclude other causes of phosphopenic and calcipenic rickets/osteomalacia. Nutritional rickets due to vitamin D deficiency, which is common in GCC countries, and XLH may sometimes coexist [11]. The diagnosis of XLH should therefore be considered if the treatment with cholecalciferol and calcium supplements fails to heal rickets in a child/adolescent with nutritional rickets/osteomalacia.

### Medical history

The diagnostic process begins with a detailed medical history, including family history, for establishing the mode of inheritance. If one of the parents is affected, then he or she may have clinical features of XLH, including disproportionate short stature, deformed legs often bearing scars from orthopedic surgical operations, abnormalities of the skull shape with frontal bossing, and loss of permanent teeth due to repeated dental abscesses and periodontal disease. He or she may also wear a hearing aid due to deafness. The affected parent may have had lifelong symptoms of fatigue and bone pain arising from osteomalacia, osteoarthritis and pseudofractures, and stiff joints from enthesopathies. Their mobility may be impaired, requiring walking aids or a wheelchair due to pain, stiff joints, and spinal stenosis. Deafness and hearing loss resembling Menière’s disease may develop in adulthood. They may also complain of headaches, dizziness, ataxia, motor and sensory symptoms secondary to craniosynostosis, Chiari type 1 malformations, and syringomyelia.

Because of the X-linked dominant inheritance, 50% of offspring of either gender from an affected mother with XLH will have the disease. If the father is affected, then 100% of his daughters but none of the sons will be affected. The diagnosis is more likely to be delayed when there is no family history of the disease, which is reported in about 30% of cases with de novo PHEX mutations [11].

An autosomal dominant inheritance pattern causing disease in offspring of either gender should raise the possibility of autosomal dominant hypophosphatemic rickets (mutation in the FGF23 gene) or vitamin D–dependent rickets type III (mutation in the CYP3A4 gene) [26].

An X-linked recessive inheritance pattern, in which only males are affected, and the mother is an asymptomatic carrier, should raise the possibility of Lowe syndrome (mutation in the OCRL-1 gene); Dent disease, type 1 (mutation in the CLCN5 gene); and Dent disease, type 2 (mutation in the OCRL-1 gene) [27–29]. In a child of either gender born to unaffected, often consanguineous parents, several possibilities may be raised. These include vitamin D–dependent rickets type IA (mutation in the CYP27B1 gene), vitamin D–dependent rickets type IB (mutation in the CYP2R1 gene), vitamin D–dependent rickets type IIA (mutation in the VDR gene), and vitamin D–dependent rickets type IIB (HNRNPC). Also, autosomal recessive hypophosphatemic rickets type IA (mutation in the DMP1 gene), autosomal recessive hypophosphatemic rickets type IB (mutation in the ENPP1 gene), autosomal recessive hypophosphatemic rickets type IC (mutation in the FAM20 gene), and hereditary hypophosphatemic rickets with hypercalciuria (HHRH; mutation in the SLC34A1 and SLC34A3 genes) may be contributory [11, 30, 31].

A positive family history of rickets/osteomalacia with nephrocalcinosis, renal stones, and chronic renal failure should raise the possibility of Dent’s disease or hereditary hypophosphatemic rickets with hypercalciuria. The possibility of renal Fanconi syndrome should be considered in a child with hypophosphatemic rickets associated with a history of polyuria, polydipsia, failure to thrive, and short stature. Hypercalciuria and nephrocalcinosis are also features of conditions that cause renal Fanconi syndrome [32, 33].

A detailed dietary history focusing on dietary calcium intake should be obtained in patients presenting with calcipenic rickets and phosphopenic rickets/osteomalacia. Lifestyle history of wearing concealing clothing and avoiding sunshine, which would limit the individual’s cutaneous vitamin D synthesis, should also be sought. Infants with severe rickets/osteomalacia arising from nutritional vitamin D and/or dietary calcium deficiency or VDDR (types IA, IB, IIA and IIC, and III) may complain of irritability, excessive sweating, muscle spasms, and hypocalcemic seizures. Adolescents with osteomalacia may complain of limb pains, myopathy, and fatigue. Infants fed on certain extensively hydrolyzed or amino acid–based formula brands may develop hypophosphatemic rickets, probably arising from poor bioavailability of phosphate [34, 35]. A detailed drug history should be obtained as certain drugs (e.g., sodium valproate, ifosfamide, cisplatin, gentamicin, and tenofovir) cause acquired renal Fanconi syndrome and associated renal phosphate wasting and phosphopenic rickets/osteomalacia [36, 37].

### Physical examination

A thorough physical examination should be performed in all cases, including detailed auxology (head circumference, standing height or length, sitting height, and body weight). Infants with XLH develop genu valgum or bowed legs upon weight-bearing. The degree of lower limb deformity should be assessed by measurement of the intermalleolar and intercondylar distances. The clinical examination may reveal the widening of ends of long bones and abnormal head shape with frontal bossing and dolichocephaly due to craniosynostosis affecting one or more cranial sutures. At presentation, the child may have disproportionate short stature. Infants may present with mild motor developmental delay due to muscle weakness. He/she may walk with a waddling gait, due to coxa vara and proximal myopathy, and an in-toeing gait due to tibial torsion. An older child may complain of easy fatigability and bone pain. Around 60% of patients with XLH have dental abscesses of deciduous and permanent teeth due to impaired mineralization of dentine and enamel, which allow bacteria to invade the pulp, leading to a tooth abscess in the absence of trauma or tooth decay [38–41].

There is considerable overlap between clinical features in patients with rickets/osteomalacia and those in patients with XLH. These features include craniotabes, delayed closure of fontanelles, frontal bossing, enlarged metaphysis, prominence of costochondral joints (rachitic rosary), Harrison’s sulcus, bowed legs, myopathy leading to motor developmental delay, and short stature arising from nutritional vitamin D and dietary calcium deficiency. Generally, the clinical features of rickets tend to be more severe in patients with VDDR (types IA, IB, IIA and IIB, and III). Those with VDDR IIA and IIB may present with partial or complete alopecia, which usually becomes apparent in the first year of life. Adolescents with osteomalacia may have genu valgum or genu varum. Chronic hypocalcemia in a patient with severe nutritional rickets and VDDR may lead to dental enamel hypoplasia, in contrast to dental abscesses in XLH. The presence of skin lesions, such as cafe-au-lait macules, should raise the possibility of hypophosphatemia secondary to fibrous dysplasia or McCune Albright syndrome. Patients with linear sebaceous nevi syndrome (Schimmelpenning syndrome) have well-demarcated linear, hairless verrucous and hyperkeratotic skin plaques, which usually follow the lines of Blaschko.

Besides the musculoskeletal and dental examination in patients with XLH, we recommend a fundoscopy and neurological examination in older adolescents, especially in those with craniosynostosis/Chiari type 1 malformations and history of headaches, dizziness, ataxia, and sensory and motor symptoms.

### Laboratory investigations

**Fig. 1:**
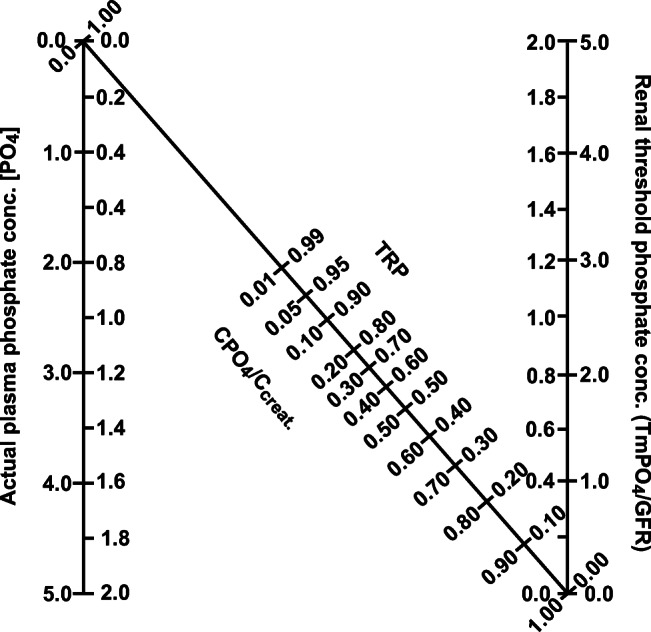
The nomogram for determination of the renal tubular threshold maximum for phosphate (TmP/GFR). The tubular reabsorption of phosphate (TRP) is calculated as described in the text*.* A line plotted from the serum concentration of phosphate on the left axis to intersect with the TRP along the central diagonal axis. Where this line intersects, the right axis represents TmP/GFR. [Reprinted from R. J. Walton and O. L. Bijvoet: Nomogram for the derivation of renal threshold phosphate concentration. Lancet 306:309 –310, 1975 (47), with permission]

In addition to recognizing clinical characteristics, laboratory investigations are crucially important in making the diagnosis of XLH. We recommend the approach outlined in Fig. 1 as a guide to laboratory investigations of a patient with rickets and osteomalacia with low serum phosphate level for age. The laboratory characteristics of inherited and acquired causes of calcipenic and phosphopenic causes of rickets and osteomalacia are detailed in Table 1.

#### Serum phosphate levels and assessment of urine phosphate excretion (Fig. 2)

Hypophosphatemia may arise from impaired dietary phosphate absorption, renal phosphate wasting, or shift of phosphate ions from the extracellular fluid into cells. The biochemical hallmark of XLH is low serum phosphate level for age, in the context of increased urinary phosphate excretion. When assessing serum phosphate level, ideally on a fasting sample, it is crucial to rely on age-appropriate normal phosphate ranges, which are higher in infant and young children than in adolescents and adults. Thus, comparing a child serum phosphate value against an adult reference may lead to misdiagnosis of phosphopenic rickets [42]. In the XLH-affected infants, serum phosphate values start to fall after birth, and in our experience, the serum phosphate level is usually below the age-appropriate lower reference value by 6 months of age. Age-appropriate reference phosphate ranges in the Australasian Association of Clinical Biochemists and the UK Pathology Harmony Group, which were derived from values in pediatric patients, are lower than those derived from prospective studies in healthy children in Canada and Northern European countries [43]. These differences in lower reference phosphate values may have an important implication in diagnosing hypophosphatemia in infants and children. Due to ethnic- and gender-related variations in serum phosphate values, a prospective study to establish pediatric reference ranges is needed in the GCC countries [44].

**Fig. 2:**
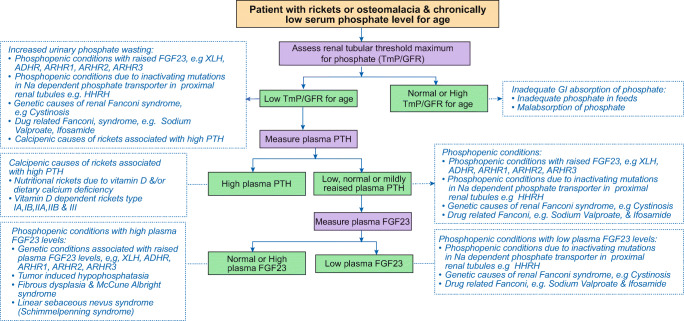
An algorithm for the assessment of a patient with rickets or osteomalacia and chronically low serum phosphate level for age, based on renal tubular threshold maximum for phosphate (TmP/GFR) and plasma levels of parathyroid hormone (PTH) and fibroblast growth factor-23 (FGF23)

In assessing phosphate handling by the kidney, it is essential to take into account the filtered load of phosphate and the amount of phosphate reabsorbed by the tubules. The most straightforward measurement of renal phosphate handling is the percentage of tubular reabsorption of phosphate (TRP), which is easily calculated from fasting serum, and fasting second-morning void urine concentrations of phosphate and creatinine (Box 1). Typically, more than 85% of the filtered load of phosphate is reabsorbed, but it approaches 100% during dietary phosphate deprivation or malabsorption. Renal tubular threshold maximum for phosphate, or maximal reabsorption rate of phosphate per unit volume of glomerular filtrate (TmP/GFR), can be determined from serum phosphate concentration and TRP, using the Walton and Bijvoet nomogram, or using a web-based tool (Box 1) [45–48]. Generally, the serum phosphate level is similar to the TmP/GFR value in the same measurement units. As for serum phosphate levels, normal TmP/GFR ranges vary according to age; infants and children have higher values than adolescents and adults [47, 49]. A patient with XLH, or other disorders leading to renal phosphate wasting, will have an inappropriately low TmP/GFR value for their prevailing low age-appropriate serum phosphate value. In contrast, a patient with low serum phosphate value for age and normal or high TmP/GFR indicates renal phosphate conservation, for example, due to inadequate dietary intake or malabsorption of phosphate.

**Table:** Box 1 Measurement of renal phosphate handling by calculation of the percentage of tubular reabsorption of phosphate (TRP) and determination of renal tubular threshold maximum for phosphate

A. Calculation of the percentage of tubular reabsorption of phosphate (TRP). $\mathrm{TRP}\%=1\hbox{-} \frac{\left[\mathrm{uP}\right]\times \left[\mathrm{sCr}\right]}{\left[\mathrm{sP}\right]\times \left[\mathrm{uCr}\right]}\times 100$ where [uP] = urine phosphate concentration (mmol/l). [sP] = serum phosphate concentration (mmol/l). [uCr] = urine creatinine concentration (mmol/l) [sCr] = serum creatinine (mmol/l).	
B. Determination of renal tubular threshold maximum for phosphate, or maximal reabsorption rate of phosphate per unit volume of glomerular filtrate (TmP/GFR): 1. From serum phosphate concentration and TRP, using the Walton Bijvoet normogram (Fig. 1)* 2. Using a web-based tool: [http://www.baspath.co.uk/calculations/renal_tubular_reabsorption_of_ph.htm]*	
*Refs. [37–40].	

As mentioned previously, hypophosphatemia is a common mechanism for all types of rickets and osteomalacia. In a patient with rickets due to impaired dietary phosphate absorption, for example, in children fed on certain extensively hydrolyzed or amino acid–based formula brands, serum phosphate level for age is low, but their TmP/GFR is normal or high for the age of the child [35]. In patients with rickets/osteomalacia arising from calcipenic causes, i.e., nutritional vitamin D and dietary calcium deficiency, or from VDDR (types IA, IB, IIA and IIB, and III), age-related serum phosphate and TmP/GFR values will be low due to secondary hyperparathyroidism. Likewise, these variables are low in patients with hereditary hypophosphatemic rickets with hypercalciuria, hereditary causes of renal Fanconi syndrome (e.g., cystinosis, tyrosinemia, galactosemia, Wilson’s disease, Dent’s disease, and Lowe syndrome), and drug-induced renal Fanconi syndrome.

#### Urine dipstick analysis

We recommend urine dipsticks analysis in all patients as glucosuria, aminoaciduria, bicarbonaturia (urine pH >7), and proteinuria might be a clue to generalized proximal tubular dysfunction that is seen in patients in conditions associated with renal Fanconi syndrome. In contrast, patients with XLH will have negative urine dipstick testing.

#### Urine calcium to creatinine ratio and protein excretion

In untreated patients with XLH, urine calcium excretion, and hence urine calcium to creatinine ratio, is normal. In contrast, urine calcium excretion is decreased in all types of calcipenic rickets and increased in hereditary hypophosphatemic rickets with hypercalciuria and in conditions associated with renal Fanconi syndrome, for example, cystinosis. Increased urinary albumin and low molecular weight protein excretion (alpha-1 microglobulin, beta-2-microalbumin, retinol-binding protein) might be a clue to Dent’s disease—the urine dipsticks test may be negative as it detects albumin rather than low molecular weight protein [50–54].

#### Serum levels of calcium

Serum levels of corrected calcium (serum calcium levels corrected for prevailing serum albumin levels) are maintained in a very tight range (2.2 to 2.65 mmol/L), with little age-related variation. Serum corrected calcium values are usually normal or at the lower end of the reference range in patients with XLH. In contrast, serum corrected calcium levels in patients with calcipenic rickets/osteomalacia arising from nutritional vitamin D and/or dietary calcium deficiency or VDDR (types IA, IB, IIA and IIB, and III) may be low, or at the lower end of the reference range. Hypercalcemia in a patient with chronically low serum phosphate levels suggests the diagnosis of hereditary hypophosphatemic rickets with hypercalciuria or primary hyperparathyroidism [55].

#### Serum levels of alkaline phosphatase

Raised serum alkaline phosphatase activity for age is a universal finding in all types of rickets. Serum alkaline phosphatase activity values vary according to the type of assay used as well as the age and gender of children and adolescents [56]. Therefore, it is essential to use age- and gender-specific reference ranges when interpreting serum alkaline phosphatase activity levels in children and adolescents. In patients with XLH, values of serum alkaline phosphatase activity for age are raised. However, it is not as high as in patients with rickets/osteomalacia arising from nutritional vitamin D and/or dietary calcium deficiency, or VDDR (types IA, IB, IIA and IIB, and III) [57].

#### Plasma parathyroid hormone levels

In untreated patients with XLH, plasma parathyroid hormone (PTH) levels are usually normal or mildly elevated due to inappropriately low serum 1,25(OH)_2_D levels [47]. Widespread vitamin D deficiency among subjects in the GCC countries may also contribute to elevated plasma PTH levels of secondary hyperparathyroidism in XLH patients. In contrast, patients with calcipenic rickets/osteomalacia arising from nutritional vitamin D and/or dietary calcium deficiency or VDDR (types IA, IB, IIA and IIB, and III) have markedly elevated plasma PTH levels [47].

#### Serum vitamin D levels

The serum level of 25OHD, a reliable serum marker of an individual’s vitamin D status, is usually normal in XLH. However, as mentioned previously, vitamin D deficiency is widespread among residents of the GCC countries, and so some patients with XLH may have low serum 25OHD values. Patients with rickets/osteomalacia due to vitamin D deficiency will have 25OHD serum levels < 30 nmol/L. [4] This chronic vitamin D deficiency or insufficiency may lead to secondary hyperparathyroidism.

Patients with VDDR1B will have low serum 25OHD levels due to biallelic mutation in the CYP2R1 gene, which encodes for the main hepatic 25-hydroxylase [30, 31, 58]. Serum 25OHD levels will also be low in subjects with VDDR type III, in whom gain of function mutation in CYP3A4 leads to vitamin D deficiency through accelerated inactivation of vitamin D metabolites [26].

In patients with XLH, serum 1,25(OH)_2_D levels are inappropriately low for the prevailing serum phosphate levels, due to high FGF23 downregulating CYP27B1 and upregulating CYP24A1 [10]. In patients with VDDR1A, a biallelic mutation in the CYP27B1, a gene encoding 25-hydroxyvitamin-d-1-α-hydroxylase, serum 1,25(OH)_2_D levels are unmeasurable, or inappropriately low for the high plasma PTH levels. In patients with hereditary hypophosphatemic rickets with hypercalciuria, low plasma FGF23 leads to high serum 1,25(OH)_2_D levels [30, 31]. Patients with VDDR type IIA and IIB have very high serum levels of 1,25(OH)_2_D levels [30, 31].

#### Plasma FGF23 levels

In patients with XLH, PHEX mutations increase plasma levels of both intact FGF23 and C-terminal FGF23 levels. However, we are aware of genetically confirmed XLH patients with plasma FGF23 levels within the normal range, which should be interpreted as inappropriately normal in the setting of hypophosphatemia [53, 54]. Patients with tumor-induced osteomalacia have very high plasma levels of FGF23 [31]. In contrast, plasma FGF23 levels are low in hypophosphatemic patients with hereditary hypophosphatemic rickets with hypercalciuria. Plasma FGF23 is low or undetectable in patients with rickets/osteomalacia arising from nutritional vitamin D and dietary calcium deficiency, VDDR (types IA, IB, IIA and IIB, and III), or in cases of rickets secondary to renal Fanconi syndrome [6, 59]. FGF23 assay is not widely available in most of the clinical facilities in the region; we recommend that its use be limited to cases where genetic diagnosis is not available or in the absence of the family history.

## Radiographs

**Fig. 3:**
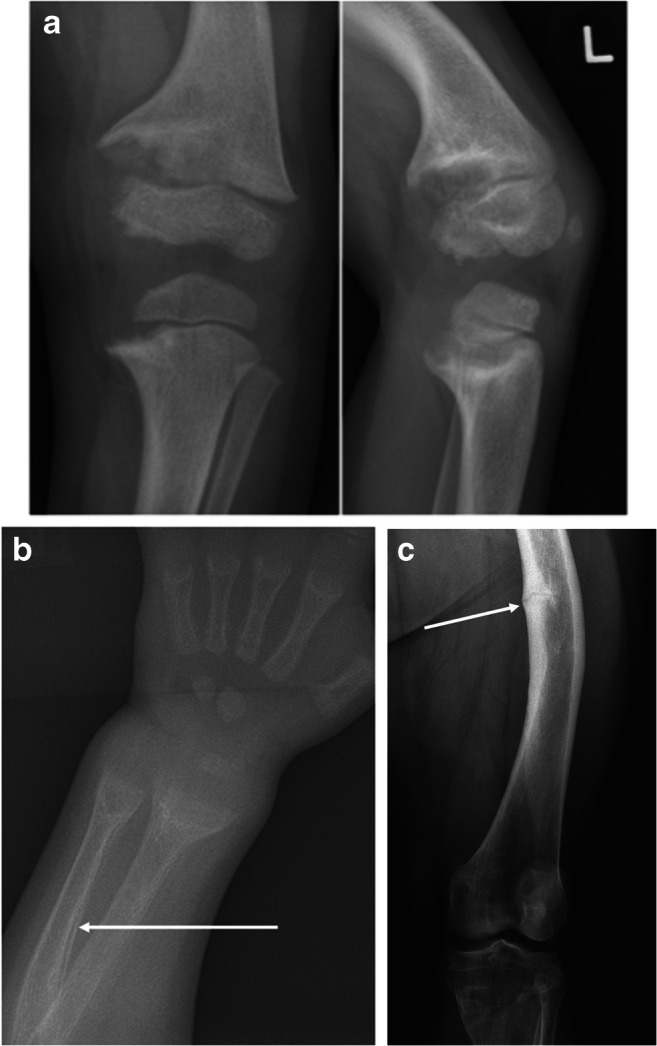
**a** Antero-posterior and lateral radiograph of the left knee in a 5-year-old boy with untreated XLH, widening, cupping, and fraying of metaphyses. Note the trabecular pattern at metaphyses and the asymmetry of the severity of the rachitic changes at the growth plate, with the medial side being more severely affected (wider and more frayed) than the lateral side. **b** Radiograph of the left wrist of a 2-year-old toddler with nutritional rickets secondary to severe vitamin D and dietary calcium deficiency. Note the florid and symmetrical widening, cupping, and fraying of metaphyses. The arrow points to periosteal reaction along diaphysis, due to severe secondary hyperparathyroidism. **c** Radiograph of the left femur from a 23-year-old man with XLH showing a medial diaphyseal pseudofracture or “Looser zone” (arrow)

In a child with XLH, radiograph of a wrist or a knee will show classical radiological changes of rickets—widening, cupping, and fraying of the metaphysis (Fig. 3a). The trabecular pattern at the distal ends of long bones often appears sclerotic. These radiological changes tend to be more marked in toddlers rather than in adolescents. An orthopedic surgeon may request standing long-leg radiographs to evaluate the mechanical axis and to plan correction of genu varum by hemi-epiphysiodesis or by femoral and tibial osteotomies. Radiological features of secondary hyperparathyroidism (Fig. 3b), such as subperiosteal bone resorption, periosteal reaction along diaphysis of long bones, and “brown tumors” seen in severe cases of calcipenic rickets, are uncommon in untreated patients with XLH. After the closure of epiphyseal growth plates, the abovementioned rachitic are not seen, but residual bowing and widening of ends of long bones persist in patients who are untreated or non-adherent to treatment. In such patients, radiographs may also show pseudofractures or “Looser zones” (Fig. 3c).

## Renal ultrasound scan

While nephrocalcinosis develops in 30–70% of XLH patients on “conventional treatment,” it is not seen at diagnosis of the condition [11]. Nephrocalcinosis with or without nephrolithiasis may be seen in renal ultrasound of patients with hypophosphatemic rickets with hypercalciuria and renal Fanconi syndrome.

## Genetic studies

Targeted next-generation sequence (NGS) gene-panels, whole-exome sequencing (WES), or whole-genome sequencing (WGS) approaches may be used in the genetic diagnosis of XLH. The use of targeted gene-panels, employing the NGS technology, has improved the clinician’s ability to quickly identify the gene responsible for inherited hypophosphatemic rickets [60, 61]. While a smaller proportion of PHEX mutation cases have duplication or deletion rather than a single nucleotide change, duplication and deletion analysis to NGS may improve the panel’s diagnostic yield [62]. In the Gulf region, genetic testing for suspected cases of XLH using NGG panels is available from some international commercial providers. These panels include several genes (DMP1, ENPP1, FGF23, PHEX, SLC34A1, FAM20C), which cause hypophosphatemic rickets. These panels are particularly useful in patients where there is no family history of XLH. The use of WES and WGS is more likely than targeted gene-panels to identify novel or rare variants, whose clinical significance may be uncertain. Several commercial genetic service providers also provide targeted NGS panels that include genes responsible for several calcipenic and phosphopenic genetic causes of rickets.

## Management of XLH in children and adolescents

We recommend that children and adolescents with XLH should be treated at centers that provide multidisciplinary care of patients with bone disorders. The team may be led by pediatric/adult endocrinologist or nephrologist with expertise in the management of bone disorders. Members of the multidisciplinary team should include orthopedic surgeons, dentists, physiotherapists, and occupational therapists. Input from a clinical geneticist, genetic counselor, audiologist, ophthalmologist and neurosurgeon, and expert pediatric radiologist may also be required for some patients. There are limited data on the transition from pediatric to adult care in the region. In many Gulf countries, the transition to adult services begins at around 14 years of age. Care transition from pediatric to adult care should be planned earlier, and for optimal care, patients should be seen in a transition clinic where care is combined between pediatric and adult medicine. There are limited data regarding the transition among patients with XLH, and a general recommendation for the transition process has been published [63].

Here we guide the medical management of XLH in children and adolescents with oral phosphate and active vitamin D analogs (conventional treatment) and with Burosumab.

## “Conventional treatment” of XLH

As raised plasma FGF23 in XLH results in urinary phosphate loss and impaired 1,25(OH)_2_D production, since the 1980s treatment of children and adolescents with XLH has involved administration of phosphate salts along with active analogs of vitamin D—calcitriol (1,25(OH)_2_D) or alfacalcidol (1-hydroxycholecalciferol). [64] Phosphate salts have an unpleasant taste and side effects: nausea, vomiting, abdominal pains, and diarrhea. There is a considerable variation in recommended doses of phosphate salts and active analogs (calcitriol or alfacalcidol) of vitamin D in published expert and consensus guidelines [3, 6, 11, 12]. Potassium or sodium-phosphate salts are given in doses of elemental phosphorus ranging from 20 to 60 mg/kg body weight, in 4 to 5 divided doses per day. At the same time, the patient is started on calcitriol at the dose of 20–30 ng/kg body weight, administered in two divided doses daily, or alfacalcidol at the dose of 30–50 ng/kg body weight daily, administered a single dose. We recommend starting phosphate salts at ~ 25% of the lower end of the recommended dose range to minimize their unpleasant side effects. As mentioned previously, secondary hyperparathyroidism is common among subjects in the Gulf region, due to widespread vitamin D deficiency. Hence it is also important to provide vitamin D supplements to maintain serum 25OH levels > 50 nmol/L.

Meticulous monitoring is essential to ensure a correct balance of phosphate salts and active analogs of vitamin D clinically and biochemically. The clinical response to treatment is assessed by monitoring growth (ideally standing and sitting height), head growth (head circumference especially in children under 5 years of age), improvement in lower limb deformity (inter-malleolar and intercondylar distances), improvement in gait and muscular function, and frequency of dental abscess. Biochemical response to treatment is assessed by measuring alkaline phosphatase and plasma parathyroid hormone 2 weeks after starting treatment and every 3-monthly after that during infancy.

Measurement of serum calcium and PTH levels is important to screen for hyperparathyroidism. Administration of pharmacological doses of phosphate slats or inadequate doses of calcitriol/alfacalcidol leads to a reduction of serum calcium, which in turn will cause secondary hyperparathyroidism. If secondary hyperparathyroidism is left untreated, then it will lead to tertiary hyperparathyroidism.

Periodic measurement of urine calcium/creatinine ratio on a spot urine sample is important to screen for hypercalciuria. A renal ultrasound scan should be performed every couple of years to screen for nephrocalcinosis. In a child, we recommend performing a radiograph of a knee 6 months after initiating treatment, to look for radiological signs of healing of rickets. During childhood, the knees’ radiographs should be performed every 1 to 2 years to guide the adjustment of medications.

In a patient with XLH with incompletely healed rickets (judged by serum alkaline phosphate level and radiographic assessment of rickets), the phosphate dose is gradually titrating up to the target dose range. Pharmacological doses of phosphate treatment often result in secondary hyperparathyroidism, and the dose of calcitriol or alfacalcidol has to be increased to normalize the plasma parathyroid level. If there is hypercalciuria, then the dose of calcitriol or alfacalcidol should be decreased until the calcium/creatinine ratio has returned to normal. In patients who are particularly prone to developing secondary hyperparathyroidism in response to phosphate therapy, adjunct treatment with cinacalcet may help suppress elevated plasma parathyroid levels and improve TmP/GFR [65, 66]. Concomitant treatment with hydrochlorothiazide may help to decrease urinary calcium excretion [67, 68].

The therapeutic goal of conventional therapy in children and adolescents is to heal rickets and osteomalacia, minimize skeletal deformities, optimize linear growth, and avoid side effects. The most notable side effects are hypercalcemia, nephrocalcinosis, nephrolithiasis, impaired renal function, secondary hyperparathyroidism, and tertiary hyperparathyroidism [69]. As mentioned previously, the goal of conventional treatment is not to normalize serum phosphate levels. In fact, unlike treatment with burosumab, it is usually not possible to achieve consistently normal fasting serum phosphate levels for the age of the child with conventional treatment. Complete healing of rickets or osteomalacia may not occur in many patients on conventional treatment—burosumab treatment led to the healing of rickets in children with XLH who had been treated with the conventional treatment for several years [70–72]. This may, in part, be due to disease severity, lack of standardization of conventional therapy, and poor adherence to treatment because of multiple daily dosing regimen, unpleasant taste, and gastrointestinal side effects [73]. Furthermore, lower limb skeletal deformities persist in many children requiring surgical correction using hemi-epiphysiodesis in the growing child or by femoral and tibial osteotomies after the end of growth. There is a general agreement that starting treatment in younger patients results in improved growth outcome. [74] A 3-year growth hormone treatment in severely short children with XLH resulted in sustained improvement of longitudinal body dimensions without progression of body disproportion [75]. However, 40% of XLH children treated with conventional therapy failed to achieve an actual height within -2 SDs of the population norm [76]. The conventional treatment improves dentin mineralization and decreases the risk of dental abscesses and severe periodontal disease. [38, 77, 78] Conventional treatment results in elevated plasma FGF23 levels, which could potentially blunt its efficacy [78, 79]. Children and adolescents with XLH are treated with conventional treatment from the time of diagnosis until growth stops. However, the treatment may also be offered to adults with symptoms of osteomalacia, such as bone pain and pseudofractures, or before or after elective orthopedic surgery [80].

## Treatment of XLH with burosumab

Burosumab is a recombinant human IgG1 monoclonal antibody that binds to and inhibits the activity of FGF23. In a phase 2 study in children with XLH, with ages ranging from 1 to 12 years, burosumab treatment normalized fasting serum phosphate levels, resulted in a decrease in serum alkaline phosphatase, and reduced rickets severity. A randomized, open-label, phase 3 study compared burosumab treatment with conventional therapy in 1- to 12-year-old children with XLH. Before enrolment into the trial, all participants had been treated with conventional therapy. The results showed that burosumab was superior to conventional therapy in normalizing serum phosphate and alkaline phosphatase levels and improving rickets severity, lower limb deformity, growth, and mobility [71].

Burosumab has been approved by the regulatory authorities in Europe, the USA, UAE, and Oman, and is under the registration process in the other GCC countries. Burosumab has been approved for the treatment of XLH by the Food and Drug Administration (FDA) in children and adults from the age of 6 months onwards and by the European Medicines Authority (EMA) in children from the age of 1 year and adolescents with growing skeletons. The practical aspects of initiation, titration, and monitoring of response to burosumab are detailed in Box 2. Short-term side effects include injection site reactions, headache, toothache, and myalgia. Burosumab is a new drug, and data on “real world,” long-term efficacy, and post-marketing pharmacovigilance should be collected through international and regional XLH registries.

**Table:** Box 2 Practical aspects of pre-treatment evaluation, initiation, uptitration, and optimization and further adjustment and monitoring of response to burosumab therapy for XLH

Stages	Actions
Pre-requisite	A. Confirmation of the diagnosis of XLH is vital before starting treatment with burosumab, and the patient’s fasting serum phosphate should be below the reference range for age.B. For patients on conventional treatment, oral phosphate and vitamin D analogs should be discontinued for at least 7 days before starting burosumab treatment.
Pre-treatment	Before initiating burosumab treatment, blood and urine samples should be collected after at least a 4-h fast for measurement of:(a) Serum/plasma concentrations of corrected calcium, phosphate, alkaline, phosphatase activity, parathyroid hormone, 25-hydroxyvitamin D. (b) Estimate tubular maximum reabsorption of phosphate (TmP/GFR). (c) Assess urine calcium/creatinine ratio.
Burosumab initiation	For infants’ age ≥ 6 months who weigh less than 10 kg: Burosumab should be initiated at the dose of 1 mg/kg body weight, rounded to the nearest 1 mg, and administered subcutaneously every 2 weeks.
	In children who weigh ≥ 10 kg: Burosumab should be initiated at a starting dose of 0.8 mg/kg body weight, rounded to the nearest 10 mg, and administered subcutaneously every 2 weeks.
Burosumab dose uptitration and optimization:	Pre-dose fasting serum phosphate levels should be measured every 2 weeks, and the dose of burosumab increased by 0.4 mg/kg body weight until fasting serum phosphate levels is at the lower end of the reference range for age. The dose of burosumab may be increased up to the maximum dose of 2 mg/kg or 90 mg, whichever is lower.
Burosumab dose adjustment:	Once a stable fasting serum phosphate level for age of the child is achieved, follow-up, including biochemical monitoring interval, may be reduced to every 3 to 6 months. If the pre-dose fasting serum phosphate is above the reference range for age, the next dose should be withheld, and the fasting serum phosphate reassessed within 4 weeks. Once the patient’s fasting serum phosphate is near or just below the reference range for age, burosumab is re-started at 50% of the previous dose.
Monitoring of response:	(a) Auxological parameters (head circumference, sitting height, and standing height), lower limb deformity (inter-malleolar and intercondylar distances), (b) dental outcomes (number of abscesses, tooth loss, root canal treatment, etc.), (c) improvement in muscle function (6-min walk test, grip strength, etc.), (d) pain scores and need for analgesics. (e) We recommend 3 to 4 monthly biochemical monitoring on fasting blood and spot urine sample: serum levels of electrolytes, urea, creatinine, corrected calcium, phosphate, alkaline phosphatase, parathyroid hormone, 25OHD, urine calcium/creatine ratio, and TmP/GFR. (f) We do not advocate measurement of plasma FGF23 levels. (g) Healing of rickets is judged by annual radiographs of the knee in a growing child. (g) A renal ultrasound should be performed every couple of years to screen for or monitor the progression of pre-existing nephrocalcinosis.

## Vitamin D supplementation and adequate dietary calcium intake

Vitamin D and/or dietary calcium deficiency will lead to elevated plasma parathyroid hormone, which will result in urinary phosphate loss and potentially negate the effect of treatment. As vitamin D deficiency is common in GCC countries [15, 74], we recommend that vitamin D deficiency be treated in all children and adolescents with XLH and that a prophylactic dose of cholecalciferol be taken daily once the vitamin D level has been normalized [4, 81, 82]. They should also be encouraged to consume sufficient quantities of calcium-rich foods, such as dairy products.

## Summary

- Chronic hypophosphatemia, which results in impaired mineralization of the growth plate in children, and osteoid both in children with rickets and in older adolescents with osteomalacia, is the common etiologic factor causing calcipenic and phosphopenic rickets/osteomalacia.
- X-linked hypophosphatemia (XLH) is caused by dominant mutations in the PHEX (phosphate-regulating endopeptidase homolog, X-linked) gene which lead to increased plasma level and expression of fibroblast growth factor-23 (FGF23), resulting in renal phosphate wasting and low serum 1,25-dihydroxyvitamin D (1,25(OH)_2_D) levels.
- XLH is the most frequent genetic cause of rickets and osteomalacia.
- Besides rickets and osteomalacia, XLH also results in disproportionate short stature, myopathy, dental abscesses, skull abnormalities (craniosynostosis, Chiari type 1 malformation with or without syringomyelia), deafness, pain, pseudofractures, fractures, enthesopathies, and spinal stenosis.
- In the absence of family history, the diagnosis of XLH is often delayed, which may be due to the rarity of the disease, variable severity, and multisystem involvement. Since all cases of rickets have low serum phosphate levels, calcipenic or other causes of phosphopenic rickets may be misdiagnosed as XLH.
- A detailed medical (and family) history, auxology, musculoskeletal examination, and appropriate investigations (radiology, biochemistry, and genetic studies) help to establish the diagnosis of XLH and exclude other causes of phosphopenic and calcipenic rickets/osteomalacia.
- The medical treatment of XLH with oral phosphate salts 4 to 5 times a day and activated vitamin D analogs (calcitriol or alfacalcidol) is known as “conventional treatment,” resulting in increased mineralization and growth and improvement in bone deformities in children and adolescents.
- The conventional medical treatment of XLH is not standardized, and except in mild cases of XLH, it does not cure or significantly improve rickets/osteomalacia. Thus, many children and adolescents require surgery to correct residual lower limb deformities.
- The efficacy of conventional therapy in adults with XLH is unclear.
- Adherence to conventional therapy is often poor due to administration frequency, poor taste, and gastrointestinal (nausea, vomiting, abdominal pain, and diarrhea) side effects. It is also associated with significant side effects: hypercalciuria, nephrocalcinosis, secondary hyperparathyroidism, and tertiary hyperparathyroidism.
- Targeted treatment with burosumab, a fully human monoclonal antibody against FGF23, results in sustained normalization of serum levels of inorganic phosphate, 1,25(OH)_2_D, and alkaline phosphatase. It results in the healing of rickets and osteomalacia. It also improves the growth rate and muscular functional capacity. Burosumab is the only available treatment addressing the underlying cause of the disease since it is inhibiting the FGF23.

## Abbreviations

1,25(OH)_2_D: 1,25-dihydroxyvitamin D
25OHD: 25-hydroxyvitamin D
ALP: alkaline phosphatase
Ca: calcium
FGF23: fibroblast growth factor-23
GCC: Gulf Cooperation Council countries
P: phosphate
PHEX: phosphate-regulating endopeptidase homolog, X-linked
PTH: parathyroid hormone
VDDR: vitamin D–dependent rickets
XLH: X-linked hypophosphatemia
